# Accidents from falls among hospitalized patients in Emergency Units: a multicenter study

**DOI:** 10.1590/0034-7167-2024-0320

**Published:** 2026-03-30

**Authors:** Bárbara Cristina Dias Giaquinto, Cristiana Mattos Camargos Oliveira, Camila Cardoso de Araújo Costa, Karina Gonçalves Dias de Barros, Bruna Figueiredo Manzo, Allana dos Reis Corrêa

**Affiliations:** I Universidade Federal de Minas Gerais. Belo Horizonte, Minas Gerais, Brazil

**Keywords:** Accidental Falls, Emergency Medical Services, Hospitalized Patients, Risk Factors, Patient Harm., Accidentes por Caídas, Servicios Médicos de Urgencia, Pacientes Hospitalizados, Factores de Riesgo, Daño del Paciente.

## Abstract

**Objectives:**

to analyze the occurrence of falls among patients hospitalized in emergency units.

**Methods:**

a cross-sectional study conducted from October 2020 to January 2021 in the emergency units of four hospitals in the city of Belo Horizonte. Sociodemographic, clinical, event-related, and post-fall data were collected and analyzed using descriptive statistics and binary logistic regression.

**Results:**

among the 89 medical records analyzed, falls were more frequent among elderly male patients, presenting with psychomotor agitation/mental confusion, occurring in the afternoon period and while on stretchers. Logistic regression revealed that patients who developed complications following the fall were associated with a higher degree of harm requiring intervention (moderate, severe, or death). Overall parameters of (OR: 19.64; p = 0.007), while only female sex related to (OR: 0.16; p = 0.037).

**Conclusions:**

in emergency settings, patients who experienced complications due to falls tended to evolve with a degree of harm that demanded more interventions and led to irreversible outcomes. Female sex was associated with the occurrence of harm not requiring intervention.

## RESUMEN

**Objetivos:**

analizar la ocurrencia de caídas en pacientes hospitalizados en unidades de emergencia.

**Métodos:**

estudio transversal realizado entre octubre de 2020 y enero de 2021 en las unidades de emergencia de cuatro hospitales de la ciudad de Belo Horizonte. Se recopilaron datos sociodemográficos, clínicos, relacionados con el evento y posteriores a la caída, los cuales fueron analizados mediante estadística descriptiva y regresión logística binaria.

**Resultados:**

entre los 89 registros médicos analizados, las caídas fueron más frecuentes en pacientes varones de edad avanzada, con agitación psicomotora/confusión mental, ocurridas en el período vespertino y mientras estaban en camillas. La regresión logística reveló que los pacientes que desarrollaron complicaciones tras la caída estuvieron asociados con un mayor grado de daño que requirió intervención (moderado, grave o fallecimiento) (OR: 19.64; p = 0.007). El sexo femenino se relacionó con un menor riesgo de daño que requiriera intervención (OR: 0.16; p = 0.037).

**Conclusiones:**

en el contexto de emergencia, los pacientes que presentaron complicaciones como consecuencia de caídas evolucionaron hacia grados de daño que demandaron más intervenciones y resultaron en desenlaces irreversibles. El sexo femenino se asoció con la ocurrencia de daños que no requirieron intervención.

## INTRODUCTION

Following the 1999 publication of the report “To Err is Human: Building a Safer Health System”, the topic of Patient Safety (PS) gained prominence within the healthcare context, among professionals, patients, and society at large. PS encompasses measures aimed at improving the quality of services as well as strategies to reduce Adverse Events (AEs), including patient falls^(1)^.

A fall is defined as “the unintentional displacement of the body to a lower level than the initial position, that is, when a person ends up on the floor or required support during movement, even if they did not reach the ground”^(2)^. It is considered a public health issue due to its consequences, such as increased care costs, physical, psychological, and social harm, and even death^(3)^. Despite being preventable, falls remain common in hospitals^(4)^.

In the context of tertiary care, falls contribute to longer hospital stays and increased patient morbidity and mortality, negatively impacting the quality of life of both the patient and their family^(5)^. They also evoke feelings of fear, guilt, anguish, and helplessness among healthcare professionals^(6)^.

Data from the *Agência Nacional de Vigilância Sanitária* (*“ANVISA”*, Brazilian Health Regulatory Agency) show that, in Brazil, between March 2014 and May 2025, falls were the fourth most reported type of incident in hospitals (172,962 - 8.9%)^(7)^. Among the sectors where patients fell within tertiary care settings is the Emergency Unit (EU).

Aiming to identify the profile of patients who experienced falls in the EU, a study conducted in an Australian tertiary hospital confirmed the occurrence of 190 falls, with an incidence of 0.63 falls per 1,000 visits. It was found that young adults aged 18 to 45, who were taking medications (antihypertensives, cardiovascular drugs, antidepressants, antipsychotics, benzodiazepines, hypnotics, and opioids), intoxicated with alcohol, presenting altered mental status, cognitive impairment following illicit drug use, sedative use, a history of falls in the past 12 months, and impaired mobility were identified as risk factors for fall episodes^(4)^.

A retrospective study conducted in a high-complexity philanthropic general hospital in southern Brazil revealed an incidence of 2.6 falls per 1,000 hospitalizations in the EU^(8)^. Meanwhile, a cross-sectional study carried out in a large southern university hospital reported a monthly fall incidence of 2.21% in inpatient units from January 2015 to July 2016. Furthermore, of the 42 reports received by the Patient Safety Center (PSC), 19.05% were from the EU^(9)^.

To date, national studies on falls occurring in the EU have presented only epidemiological data. However, they have not analyzed risk factors, post-fall management, nor the complications and outcomes resulting from the event, therefore justifying the relevance of this study.

It is believed that understanding the additional intrinsic elements of fall events will provide stronger scientific support to professionals working in emergency departments. Moreover, it will foster the development of more targeted and consistent strategies aimed at predictive care for hospitalized patients at risk of falling in this context.

## OBJECTIVES

To analyze the occurrence of falls among patients hospitalized in emergency units.

## METHODS

### Ethical Aspects

The study was conducted in accordance with national and international ethical guidelines for research involving human subjects and was approved by the Ethics Committee of the proposing institution and its co-participants. It is worth noting that, under the guidance of the Ethics and Research Committee of the proposing institution, both the Data Use Consent Form and the Informed Consent Form were developed, reviewed, and approved as part of the research project. However, during the project’s review process at the participating sites, all institutions requested the exclusion of the latter as a prerequisite for initiating data collection.

### Study Design, Period, and Location

This is a cross-sectional study guided by the Strengthening the Reporting of Observational Studies in Epidemiology (STROBE) tool^(10)^, aimed at analyzing falls among patients hospitalized in emergency departments.

The study was conducted from October 2020 to January 2021 in four large general hospitals located in the city of Belo Horizonte, Minas Gerais, all of which had active Patient Safety Centers. The average number of monthly emergency unit visits at each hospital was as follows: Hospital 1 - 650 visits/month; Hospital 2 - 8,000 visits/month; Hospital 3 - 5,000 visits/month; and Hospital 4 - 6,800 visits/month.

### Population; Inclusion and Exclusion Criteria

The target population consisted of medical records of adult patients who experienced falls in the emergency unit and whose incidents were reported to the PSC of the participating institutions.

Given that falls remain underreported in tertiary care settings^(9)^, and due to limited prior access to the total number of cases in each institution - most records being manual - and the absence of a theoretical framework to support sample size calculation, it was decided to investigate all fall incidents that occurred in the EUs of the selected institutions during the study period.

Included were medical records of patients aged 18 years or older who experienced a fall in the EU and whose incidents were reported to the PSC between April 2013 and December 2019. The study period was defined based on the publication date of Ordinance No. 529/2013, which established the *Programa Nacional de Segurança do Paciente* (National Patient Safety Program)^(11)^. Excluded were medical records that did not confirm the occurrence of a fall in the EU. In total, 89 medical records comprised the study population.

### Study Protocol

Initially, appointments were scheduled with each PSC to access the fall incident reports recorded during the study period.

Once the notification data were obtained, the medical records of patients who had experienced falls were retrieved. In one hospital, since the PSC had access to these records, it was possible to consult the electronic medical records within the physical space of that department. In two other institutions, which used physical records, a list of patient names and registration numbers was prepared in advance and sent to the respective *Serviço de Arquivos Médicos e Estatística* (*“SAME”*, Medical Records and Statistics Services) for retrieval and availability. In these cases, data collection was carried out by prior appointment and under the direct supervision of staff from the respective departments. In another location, since one of the authors was employed there during the data collection period, access to the medical records was granted through her personal login credentials to the operating system, in a reserved room and during non-working hours.

A data collection instrument developed by the authors was used, consisting of variables based on national and international scientific literature related to the topic of falls in emergency units^(4,12,13)^. Subsequently, the instrument was evaluated by a panel of experts with recognized expertise in Patient Safety.

The variables included in the instrument were: sociodemographic data (sex, age, and marital status), clinical data (comorbidities and lifestyle habits), risk factors (history of falls, severe pain, nervous system disorders, musculoskeletal system impairment, cognitive changes, presence of mental disorders, sensory system impairment, and assistive devices), post-fall management (medical evaluation, computed tomography, and indication for physical restraint), complications (abrasions, lacerations, hematomas, mild, moderate, or severe traumatic brain injury, fractures, and death), degree of harm (none, mild, moderate, severe, and death), and outcomes (discharge, outpatient referral, transfer to another hospital, death due to fall-related complications, and death due to clinical complications resulting from the fall).

### Analysis of Results and Statistics

The data were transcribed using double entry into a pre-structured database created in the electronic software EpiData^®^ version 3.1. This database was then exported to the Statistical Software for Professionals (STATA) version 15.0. Descriptive statistical analysis was performed, calculating absolute and relative values for categorical variables. For the quantitative variable “age”, asymmetry was assessed using the Shapiro-Wilk test^(14)^, and the median, minimum, and maximum values were reported.

The association between sociodemographic and clinical variables and the occurrence of harm was analyzed. For this purpose, two groups were considered: harm not requiring intervention (no harm or mild harm) and harm requiring intervention (moderate harm, severe harm, or death). Bivariate analysis was conducted using Pearson’s Chi-square test and Fisher’s Exact test, followed by binary logistic regression (odds ratio and confidence interval [CI] > 95%).

## RESULTS

More than half of the falls (62.8%) occurred in two hospitals that are reference centers in the Urgent and Emergency Care Network of the capital of Minas Gerais and its metropolitan region: Hospital 4 (40 cases - 44.9%) and Hospital 2 (16 cases - 17.9%). The remaining incidents took place in general hospitals within the municipality: Hospital 3 (25 cases - 28.0%) and Hospital 1 (8 cases - 8.9%).

Of the 89 medical records of patients who experienced falls in the EU, more than half were male (58.4%) and belonged to the age group of 60 years or older (59.5%). The median age was 64 years, with a minimum of 21 and a maximum of 88 years. Marital status was recorded in less than half of the cases (49.4%). Sociodemographic and clinical data of the patients who experienced falls in the respective EUs are presented in Table 1.

**Table 1:** Distribution of sociodemographic and clinical data of patients who experienced falls in emergency departments, 2013-2019, Belo Horizonte, Minas Gerais, Brazil

Variables	n	%
**Sex**		
Female	37	41.6
Male	52	58.4
**Age (years)**		
18-25	6	6.7
26-35	1	1.1
36-45	15	16.8
46-59	14	15.7
60 years or older	53	59.5
**Marital Status**		
Lives without a partner	30	68.2
Lives with a partner	14	31.8
**Comorbidities**		
Systemic arterial hypertension	42	47.2
Diabetes mellitus	26	29.2
Heart disease	23	25.8
Ischemic stroke	6	6.7
**Lifestyle Habits**		
Alcohol use	22	24.7
Tobacco use	18	20.2
Illicit drug use	7	7.8

The data regarding the time of day when the fall occurred and the type of fall are presented in Table 2.

**Table 2:** Distribution of time of day and type of fall occurring in emergency departments, 2013-2019, Belo Horizonte, Minas Gerais, Brazil

Variables	n	%
**Time of Day When the Fall Occurred**		
Afternoon	25	29.4
Early morning	24	28.2
Morning	21	24.7
Night	15	17.6
**Type of Fall**		
Fall from stretcher, bed, wheelchair, or chair/armchair	63	73.2
Fall from standing height	15	17.4
Slip	6	7.0
Fainting	1	1.1
Loss of balance	1	1.1

It is noteworthy that in 4.49% of the medical records, the time of the event was not documented, and in 3.37% the type of fall was not specified. It was found that in 1.12% of the reviewed documents, three falls occurred during the patient’s stay in the EU, and in 4.49% it was recorded that patients fell twice within a 24-hour period.

Regarding the most frequent type of fall, the majority (68.2%) fell from a stretcher, followed by falls from a bed (25.4%), wheelchair (3.2%), and chair/armchair (3.2%).

As for falls from stretchers, 9.3% occurred while the patient was lying in the supine position, 14.0% happened during patient mobilization, and in 7.0% of cases, patients experienced a seizure while on the furniture.

Factors related to patients’ clinical conditions, as well as those inherent to the healthcare process as documented in the reviewed medical records, are presented in Table 3.

**Table 3:** Distribution of clinical conditions and healthcare process factors of patients who experienced falls in emergency departments, 2013-2019, Belo Horizonte, Minas Gerais, Brazil

Variables	n	%
**Clinical Conditions** Mental confusion / psychomotor agitation	37	41.6
Gait disturbance	22	24.7
Cognitive impairment	16	18.0
Depression	14	15.7
Balance disorder	13	14.6
History of falls	10	11.2
Anxiety	8	9.0
Hearing impairment	5	5.6
Visual impairment	5	5.6
Dementia	1	1.1
**Healthcare Process** Peripheral venous access	66	74.2
Indwelling urinary catheter	5	5.6
Nasogastric feeding tube	2	2.2

Regarding fall risk assessment, it was observed that in 42.7% of the medical records, this evaluation was documented on the patient’s admission date. In 47.2%, the date of the fall event was recorded. However, in 3.4% of the reviewed documents, there was no notation regarding the assessment.

Among the actions taken by healthcare professionals following the fall were: cranial computed tomography (20.2%), clinical evaluation (18.0%), fall prevention instructions (14.6%), mechanical restraint of upper limbs (9.0%), and peripheral vein puncture (4.5%).

The complications and outcomes of hospitalization for patients who experienced falls in the studied EUs are presented in Table 4.

**Table 4:** Distribution of complications and outcomes of patients who experienced falls in emergency departments, 2013-2019, Belo Horizonte, Minas Gerais, Brazil

Variables	n	%
**Complications due to the fall** Abrasion	11	12.3
Laceration	11	12.3
Mild traumatic brain injury	9	10.1
Hematoma	7	7.8
Death	5	5.6
Fracture	4	4.4
Moderate traumatic brain injury	2	2.2
Severe traumatic brain injury	1	1.2
**Outcome** Discharge	52	58.4
Outpatient referral	21	23.5
Transfer to another institution	9	10.1
Death due to fall-related complications	5	5.6
Death to the clinical conditions	2	2.4

Regarding the distribution of the degree of harm in the studied population, no records indicated the occurrence of severe harm. In 14.6% of the analyzed medical records, this information was not documented. Among the remaining cases, 50.0% of patients presented no harm, 31.5% had mild harm, 11.8% moderate harm, and 5.6% resulted in death.

According to binary logistic regression analysis, patients who developed complications resulting from the fall were 20 times more likely to experience harm requiring intervention. Additionally, female patients were 6.25 times less likely (1/0.16) to experience moderate or severe harm, or death (Table 5).

**Table 5:** Factors associated with the occurrence of harm requiring intervention (moderate harm, severe harm, or death) in patients who experienced falls in emergency departments, 2013-2019, Belo Horizonte, Minas Gerais, Brazil

Variables	OR^*^	CI^**^ 95% Lower	CI^**^ 95% Upper	value *p*
Fall-related complications	19.64	2.21	174.8	0.007
Female Sex	0.16	0.03	0.9	0.037

* OR - Odds Ratio;

** CI - Confidence interval.

## DISCUSSION

Regarding the sociodemographic profile, a higher frequency was observed among male patients. Falls in this group occur because men generally request less help or assistance with daily activities, even when admitted to healthcare institutions^(15)^. Furthermore, for individuals of this gender, being ill is often perceived as a sign of weakness rather than a change in health status^(16)^.

The population aged 60 years or older was the most affected in this study. A qualitative study conducted in the state of Paraná, which investigated elderly women’s knowledge about fall prevention, revealed that participants recognized factors such as weakness, chronic illnesses, visual and cognitive impairments, along with the frailties and limitations inherent to aging, as contributors to the occurrence of falls^(17)^. It is important to highlight that cognitive frailty in older adults is a predictive factor for falls^(12)^. Moreover, the growth of this demographic profile will lead to an increase in hospital admissions, thereby contributing to a higher incidence of falls^(18)^.

In terms of clinical aspects, it was found that individuals who fell in the EU were smokers and consumed alcohol. Alcohol and tobacco are contributing factors to falls, as they lead to deterioration of bone mass and muscle tone, resulting in increased muscle stiffness^(19)^. Additionally, alcohol consumption depresses the central nervous system, causing ataxic gait, balance disturbances, loss of reflexes, and mental confusion^(19)^.

With regard to comorbidities, systemic arterial hypertension was the most prevalent, corroborating findings from previous studies conducted in emergency departments^(5,9)^. The presence of this condition leads to thickening of the arterial wall and endothelial dysfunction, followed by increased stiffness and reduced vascular compliance. Furthermore, fragmentation and disorganization of elastin fibers, which are replaced by collagen, facilitate calcium ion deposition, impacting osteoarticular function^(20)^. These changes impair body balance, making hypertensive individuals more susceptible to falls^(20)^.

Regarding the time of day when falls occurred, the afternoon and early morning hours were the most frequent. During the early morning, staffing levels at the bedside tend to decrease, and fewer individuals circulate within the hospital environment, resulting in reduced direct patient care. This compromises patient monitoring and, consequently, increases the risk of falls during this period^(6)^. The presence of a companion for patients at high risk of falling may serve as a key strategy for preventing such events in EU^(21)^.

Falls were more frequent among individuals lying on stretchers, as most patients admitted to EU are accommodated on this type of furniture. Prolonged immobility in a single position can lead to muscle stiffness and weakness, prompting patients to reposition themselves, which increases the risk of falling^(13)^. This finding may also reflect the high demand for public tertiary care facilities in Brazil, where EU serve as the primary entry point into this level of care. Notably, the study was conducted in two hospitals that are part of the urgent and emergency care network of the capital city of Minas Gerais and its metropolitan region^(21)^.

Psychomotor agitation and mental confusion are well-documented risk factors for patient falls^(22)^. Patients hospitalized in EU often present with complex clinical conditions and multiple comorbidities that may contribute to such symptoms^(23)^. The environment in these units is frequently hectic, with excessive noise and conversations, compounded by the slow adaptation process to the hospital setting, particularly among elderly patients, which can trigger agitation or confusion and heighten the risk of falling^(16)^.

Regarding fall prediction, risk assessment should be performed upon patient admission, daily, and continue until discharge from the healthcare institution^(11)^. However, the present study reveals discrepancies when compared to the literature. An exploratory study conducted in 2016 across eight emergency care units in Paraná found that, among 377 patients, none were assessed or flagged for fall risk^(22)^. Considering the profile of individuals treated in the EU, as well as the primary demands addressed in these settings, it is evident that fall risk prediction should occur both during triage and at other points of care, in support of a safe and directive prevention plan.

In Brazil, the Morse Fall Scale (MFS) is widely used and recommended as a fall risk predictor⁽^2^⁾. However, it appears to offer limited effectiveness in accurately assessing fall risk and in guiding targeted care for patients hospitalized in emergency departments. The use of MFS in this setting warrants further discussion.

Following a fall event, patients underwent computed tomography and clinical evaluation. Medical assessment after a fall should be immediate to determine the patient’s health status, identify potential complications, and, if necessary, initiate urgent and emergency clinical interventions^(24)^. The biomechanics of a fall involve bodily impact against surfaces, and depending on the force exerted, may result in injuries and trauma that do not always present with visible signs or symptoms. Therefore, imaging exams such as computed tomography should be performed as a complement to medical evaluation^(25)^.

Lacerations and abrasions were the most frequent complications observed in the study. A descriptive and retrospective American study conducted in an EU revealed that, among 57 falls, 5.4% resulted in abrasions^(26)^. Another U.S.-based study in the same context showed that, out of 190 events, lacerations occurred in 17.9% of cases. These injuries are among the most common because individuals often attempt to protect themselves during a fall. Typically, such wounds are concentrated in the upper and lower limbs^(27)^.

In the present study, five patients died as a result of the fall. It is important to note that the mortality rate among elderly individuals due to falls in hospital settings increases by approximately 15% per year⁽^2 8^⁾. Data from DATASUS in 2019 indicated that 342 adult patients died in Brazilian hospitals due to falls from beds. A study conducted in the *Distrito Federal* (capital of Brazil) found that, between 1996 and 2017, a total of 2,828 elderly individuals died as a result of falls, with 94.8% of these deaths occurring in tertiary care settings^(29)^.

Regarding the documentation of harm severity, most cases involved no harm or mild harm. Among the notifications received by the regulatory agency, nearly all (169,742 - 98.1%) included a record of the degree of harm. Mild harm was the most frequent (86,178 - 49.8%), while death was the least reported (429 - 0.25%)^(7)^. These findings may be related to limited awareness among healthcare professionals regarding the concept of falls and their potential psychological and social consequences.

Regarding the association between sociodemographic and clinical variables and the degree of harm, female patients were less likely to experience harm requiring intervention (moderate, severe, or death). It is understood that women tend to follow health guidance more closely and seek medical care earlier when necessary.

Patients who developed complications as a result of the fall were more likely to experience harm requiring intervention. One study showed that fall-related injuries, including cranial trauma, can worsen a patient’s clinical condition and lead to irreversible outcomes^(30)^. In this context, even when minimal interventions are required, a fall can significantly impact the patient’s health status during hospitalization.

Patients admitted to EU are expected to receive optimal care with high chances of survival. However, the occurrence of a preventable event such as a fall introduces complications that were previously absent or unexpected. Therefore, fall prevention remains a critical challenge in minimizing complications and unfavorable, and sometimes irreversible, outcomes^(21)^.

Analyzing the context and nuances of falls in EU is essential for preventing their occurrence, especially considering that patients in these settings often present with complex clinical conditions and deteriorating health. This reinforces the relevance of the present study.

### Study limitations

Among the limitations, one can highlight the current lack of validated instruments to assess the occurrence of falls in emergency departments. Another issue was the absence of documentation regarding the degree of harm, type of fall, and fall risk assessment at admission and on the day of the event in the incident reporting forms of some institutions. This led to data loss and limited the ability to perform more robust statistical analyses. Additionally, underreporting of fall events in this setting restricted the sample size and, consequently, the generalizability of the results.

### Contributions to the Fields of Nursing, Health, and Public Policy

The findings of this study are believed to offer valuable contributions to nursing education, research, and clinical practice. In education, they may encourage discussions and expand the learning of future nurses regarding fall prevention, with particular attention to the elderly population, which was the most affected by the event and is more likely to be admitted to emergency departments.

In research, the study may stimulate the development of more robust designs that explore best practices for fall prevention in this setting. In clinical practice, the findings may support the implementation of specific fall prevention protocols in EU, promoting more directive, safe, and consistent care aimed at preventing falls in this sector.

## CONCLUSIONS

The study identified that falls in EUs occurred most frequently during the afternoon, among male and elderly patients. Being accommodated on a stretcher and presenting with psychomotor agitation or mental confusion were the most common factors in this context. Fall risk assessment was not routinely performed at patient admission or on the day of the adverse event. The most commonly applied post-fall interventions included computed tomography, clinical evaluation, and fall prevention guidance. Regarding complications, lacerations and abrasions were the most prevalent. Hospital discharge was the predominant outcome, although five patients died as a result of the adverse event. It was found that experiencing complications due to a fall was associated with degrees of harm requiring more interventions and leading to irreversible outcomes. Female sex was associated with the occurrence of harm not requiring intervention.

Analyzing falls in this context requires greater awareness and investment in tertiary care settings, in order to promote evidence-based strategies aimed at reducing the occurrence of such events to the lowest acceptable level.

## Data Availability

The research data are available in a repository: https://doi.org/10.48331/SCIELODATA.UI4WW6.
