# Supplementary material for: Accidents from falls among hospitalized patients in Emergency Units: a multicenter study
Source: Rev Bras Enferm. 2026 Mar 30;79:e20240320. doi: 10.1590/0034-7167-2024-0320 (PMC13038222; doi:10.1590/0034-7167-2024-0320)
Supplement: Translation

# Translation: Acidentes por quedas de pacientes internados em unidades de Pronto- Socorro: estudo multicêntrico

**DOI:** 10.1590/0034-7167-2024-0320pt

**Authors:** Bárbara Cristina Dias Giaquinto, Cristiana Mattos Camargos Oliveira, Camila Cardoso de Araújo Costa, Karina Gonçalves Dias de Barros, Bruna Figueiredo Manzo, Allana dos Reis Corrêa

**Affiliations:** I Universidade Federal de Minas Gerais. Belo Horizonte, Minas Gerais, Brasil

**Keywords:** Acidentes por Quedas, Serviços Médicos de Emergência, Pacientes Internados, Fatores de Risco, Dano ao Paciente.

## Abstract

**Objetivos:**

analisar a ocorrência de quedas em pacientes internados em unidades de pronto-socorro.

**Métodos:**

estudo transversal, realizado entre outubro de 2020 a janeiro de 2021, em prontos-socorros de quatro hospitais de Belo Horizonte. Foram coletados dados sociodemográficos, clínicos, do evento e do pós-evento queda, e analisados através de estatística descritiva e regressão logística binária.

**Resultados:**

dos 89 prontuários analisados, as quedas foram mais frequentes em idosos do sexo masculino, com agitação psicomotora/confusão mental, no período vespertino e na maca. À regressão, foram relacionados ao grau de dano com necessidade de intervenção (moderado, grave ou óbito) pacientes que após a queda evoluíram com complicações advindas do evento (OR:19,64; p=0,007); sexo feminino (OR: 0,16; p=0,037).

**Conclusões:**

em pronto-socorro, pacientes com complicações em decorrência da queda evoluíram com grau de dano que demanda mais intervenções e desfecho irreversível. Sexo feminino foi associado à ocorrência de danos sem necessidade de intervenção.

## INTRODUÇÃO

Após a publicação em 1999 do Relatório *To Err is Human: building a safer health system*, a temática Segurança do Paciente (SP) ganhou notoriedade junto ao contexto saúde, aos profissionais, pacientes, bem como para a sociedade em geral. A SP abrang e medidas em prol de melhorias da qualidade dos serviços bem como estratégias para redução de Eventos Adversos (EA), entre eles a queda de pacientes^(1)^.

A queda é definida como “o deslocamento não intencional do corpo para um nível inferior à posição inicial, ou seja, quando a pessoa se encontra no chão ou necessitou de amparo durante um deslocamento, mesmo que ainda não esteja no solo”^(2)^. É considerada um problema de saúde pública devido às suas consequências, como oneração assistencial, danos físicos, psicológicos, sociais, até a morte^(3)^. Porém, mesmo sendo evitável, ainda é comum em hospitais^(4)^.

No contexto da atenção terciária, a queda favorece o aumento do tempo de permanência e a morbimortalidade dos pacientes, altera a qualidade de vida de quem cai e de seus familiares^(5)^, além de causar sentimentos de medo, culpa, angústia e impotência nos profissionais de saúde^(6)^.

Dados da Agência Nacional de Vigilância Sanitária (ANVISA) mostram que, no Brasil, no período de março de 2014 a maio de 2025, a queda foi o quarto tipo de incidente mais notificado por hospitais (172.962 - 8,9%)^(7)^. Dentre os setores nos quais o paciente caíram na atenção terciária está o pronto-socorro (PS).

Com o objetivo de identificar o perfil de pacientes que sofreram quedas no PS, um estudo realizado em um hospital terciário australiano confirmou a ocorrência de 190 quedas e uma incidência de 0,63 quedas/1.000 atendimentos. Verificou-se que jovens entre 18 a 45 anos, em uso de medicamentos (anti-hipertensivos; cardiovasculares; antidepressivos; antipsicóticos; benzodiazepínicos; hipnóticos e opioides), intoxicados com álcool, com estado mental alterado, com comprometimento cognitivo pós uso de drogas ilícitas, uso de sedativos, histórico de quedas nos últimos 12 meses e mobilidade prejudicada foram fatores de risco para os episódios de queda^(4)^.

Pesquisa retrospectiva realizada num hospital geral de alta complexidade e filantrópico do Sul do Brasil revelou a incidência de 2,6 quedas para cada 1.000 internações em PS^(8)^. Já o estudo transversal realizado em um hospital universitário sulista de grande porte apontou incidência de 2,21% de quedas mensais nas unidades de internação, de janeiro de 2015 a julho de 2016. Além disso, das 42 notificações que o Núcleo de Segurança do Paciente (NSP) recebeu, 19,05% eram do PS^(9)^.

Verifica-se que, até o momento, os estudos nacionais sobre queda ocorridas em PS apontam somente dados epidemiológicos. No entanto, não analisaram os fatores de risco, as condutas após a queda, bem como as complicações e desfechos advindos do evento, justificando, assim, a relevância deste estudo.

Acredita-se que o conhecimento sobre os demais elementos intrínsecos ao evento queda trará maior respaldo científico aos profissionais inseridos em PS. Ainda, contribuirá para o fomento de estratégias mais diretivas e consistentes em prol de assistência preditiva de quedas aos pacientes internados nesse contexto.

## OBJETIVOS

Analisar a ocorrência de quedas em pacientes internados em unidades de pronto-socorro.

## MÉTODOS

### Aspectos éticos

O estudo foi conduzido em conformidade com as diretrizes éticas nacionais e internacionais referentes à pesquisa com seres humanos, sendo aprovado pelo Comitê de Ética da instituição proponente e coparticipantes. Vale ressaltar que, por diligência do Comitê de Ética e Pesquisa da instituição proponente, foram construídos, apreciados e aprovados junto ao projeto de pesquisa o Termo de Consentimento de Utilização de Dados e o Termo de Consentimento Livre e Esclarecido. Entretanto, durante a apreciação do projeto nas localidades pesquisadas, todas solicitaram a exclusão do último como critério para o início da coleta.

### Desenho, período e local do estudo

Trata-se de um estudo transversal, norteado pela ferramenta *Strengthening the Reporting of Observational Studies in Epidemiology* (STROBE)^(10)^, para analisar as quedas de pacientes internados em PS.

O estudo foi desenvolvido de outubro de 2020 a janeiro de 2021 em quatro instituições hospitalares gerais e de grande porte de Belo Horizonte/MG, todas com Núcleos de Segurança do Paciente atuantes, e com a seguinte média de atendimentos no PS: hospital 1 - 650 atendimentos/mês; hospital 2: 8.000 atendimentos/mês; hospital 3: 5.000 atendimentos/mês e hospital 4: 6.800 atendimentos/mês.

### População; critérios de inclusão e exclusão

A população-alvo foi composta por prontuários de pacientes adultos que sofreram queda em PS e cujo evento tenha sido notificado junto ao NSP das instituições pesquisadas.

Por se tratar de um evento ainda subnotificado na atenção terciária^(9)^, a limitação de acesso prévio ao número total de casos de cada instituição, devido à maioria dos registros serem manuais e a ausência de referencial teórico que sustentasse um cálculo amostral, optou-se por investigar todas as quedas ocorridas em PS das instituições pesquisadas durante o período-alvo.

Foram incluídos os prontuários de pacientes com idade maior ou igual a 18 anos que sofreram queda no PS e que foram notificados no NSP da instituição no período de abril de 2013 a dezembro de 2019. A determinação do período do estudo justifica-se pela data de publicação da Portaria n. 529/2013 que instituiu o Programa Nacional de Segurança do Paciente^(11)^. Foram excluídos os prontuários que não confirmavam o evento da queda em pacientes do setor pesquisado. Ao final, 89 prontuários compuseram a população do estudo.

### Protocolo do estudo

Primeiramente, houve o agendamento com cada NSP das datas para o acesso às notificações de quedas registradas no período do estudo.

De posse dos dados das notificações, ocorreu a busca dos prontuários dos pacientes que sofreram queda. Em um hospital, pelo fato de o NSP ter acesso a estes, foi possível consultar os prontuários eletrônicos no espaço físico do referido setor. Em outras duas instituições, por disporem de prontuários físicos, foi elaborada previamente uma lista com os nomes e registros dos pacientes e enviada aos respectivos Serviço de Arquivos Médicos e Estatística (SAME) para localização e disposição dos mesmos. Nessas, a coleta ocorreu mediante agendamento prévio e sob a supervisão direta de colaboradores dos respectivos setores. Já em outra localidade, pelo fato de uma das autoras ser funcionária durante o período da coleta, o acesso aos prontuários foi feita por meio de *login* e senha pessoal do sistema operacional, em sala reservada e horários díspares ao laboral.

Foi utilizado para a coleta um instrumento elaborado pelas autoras, composto por variáveis baseadas na literatura científica nacional e internacional relacionada à temática quedas em PS^(4,12,13)^. Posteriormente, o mesmo foi avaliado por um grupo de especialistas com expertise em Segurança do Paciente.

As variáveis contempladas no instrumento foram: dados sociodemográficos (sexo, idade e estado civil), dados clínicos (comorbidades e hábito de vida), fatores de risco (histórico de queda, dor intensa, alterações do sistema nervoso, comprometimento do sistema musculoesquelético, alterações cognitivas, presença de transtornos mentais, comprometimento do sistema sensorial e dispositivos assistenciais), condutas pós queda (avaliação médica, realização de tomografia computadorizada e indicação de contenção), complicações (escoriações, ferimentos corto-contusos, hematomas, traumatismo crânio encefálico leve, moderado ou grave, fratura e óbito), grau do dano (nenhum, leve, moderado, grave e óbito) e desfechos (alta, encaminhamento ambulatorial, transferência para outro hospital, óbito por complicações da queda e óbito por complicações clínicas advindos da queda).

### Análise dos resultados e estatística

Os dados foram transcritos, por meio de dupla digitação, ao banco de dados previamente elaborado no programa eletrônico EpiData^®^ - versão 3.1. Este foi exportado para o programa *Statistical Software for Professional* (STATA) versão 15.0. Procedeu-se a análise estatística descritiva, sendo calculados os valores absolutos e relativos para as variáveis categóricas. Para a variável quantitativa idade, a assimetria foi verificada por meio do teste *Shapiro-Wilk* ^(14)^ sendo apresentada a mediana, mínimo e máximo.

Optou-se por analisar a associação entre as variáveis sociodemográficas e clínicas com a ocorrência de danos. Para isso considerou-se dois grupos: danos sem necessidade de intervenções (nenhum dado ou dano leve) e danos com necessidade de intervenções (dano moderado, dano grave ou óbito). Procedeu-se análise bivariada por meio dos testes Qui-quadrado de Pearson e o Teste Exato de Fisher e, posteriormente, regressão logística binária (*odds ratio* e índice de confiança (IC) > 95%).

## RESULTADOS

Mais da metade das quedas (62,8%) ocorreu em dois hospitais que são referências da Rede de Atenção às Urgências e Emergências da capital mineira e região metropolitana: hospital 4 (40 - 44,9%) e hospital 2 (16 - 17,9%). Já os demais eventos ocorreram nas instituições gerais do município: hospital 3 (25 - 28,0%) e hospital 1 (8 - 8,9%).

Dos 89 prontuários de pacientes vítimas de quedas em PS, mais da metade era do sexo masculino (58,4%) e pertencia a faixa etária de 60 anos ou mais (59,5%). A mediana de idade foi de 64 anos, com o mínimo de 21 anos e máximo de 88 anos. Em menos da metade (49,4%) havia o registro do estado civil. Dados sociodemográficos e clínicos dos pacientes que sofreram quedas nos referidos PS estão descritos na Tabela 1.

**Tabela 1:** Distribuição dos dados sociodemográficos e clínicos dos pacientes que sofreram queda em unidades de pronto-socorro, no período 2013-2019, Belo Horizonte, Minas Gerais, Brasil

Variáveis	n	%
**Sexo**		
Feminino	37	41,6
Masculino	52	58,4
**Idade (anos)**		
18 - 25	6	6,7
26 - 35	1	1,1
36 - 45	15	16,8
46 - 59	14	15,7
60 anos ou mais	53	59,5
**Estado Civil**		
Vive sem companheiro(a)	30	68,2
Vive com companheiro(a)	14	31,8
**Comorbidades**		
Hipertensão arterial sistêmica	42	47,2
Diabetes mellitus	26	29,2
Doença cardíaca	23	25,8
Acidente vascular encefálico isquêmico	6	6,7
**Hábitos de vida**		
Etilismo	22	24,7
Tabagismo	18	20,2
Usuário de drogas ilícitas	7	7,8

Os dados referentes ao período do dia em que ocorreu a queda e o tipo de queda estão apresentados na Tabela 2.

**Tabela 2:** Distribuição do período e tipo de queda ocorridos em unidades de pronto-socorro, no período 2013-2019, Belo Horizonte, Minas Gerais, Brasil

Variáveis	n	%
**Período em que ocorreu a queda**		
Vespertino	25	29,4
Madrugada	24	28,2
Matutino	21	24,7
Noturno	15	17,6
**Tipo de queda**		
Queda da maca, cama, cadeira de rodas e cadeira/poltrona	63	73,2
Queda da própria altura	15	17,4
Escorrego	6	7,0
Desmaio	1	1,1
Perda de equilíbrio	1	1,1

Ressalta-se que em 4,49% dos prontuários não foi registrado o período em que ocorreu o evento, e em 3,37% não foi apontado o tipo de queda. Verificou-se que em 1,12% dos documentos estudados constava a ocorrência de três quedas durante a permanência no PS, e em 4,49% estava anotado que pacientes caíram duas vezes no período de 24 horas.

No que se refere ao tipo de queda mais frequente, a maioria (68,2%) caiu da maca, seguido por queda da cama (25,4%), da cadeira de rodas (3,2%) e da cadeira/poltrona (3,2%).

Quanto à queda da maca, 9,3% quebraram com o paciente deitado e em decúbito dorsal, 14,0% viraram durante a mobilização do paciente e em 7,0% dos casos os pacientes tiveram crise convulsiva sobre a mobília.

Os fatores relativos às condições clínicas dos pacientes, bem como inerentes ao processo de atenção à saúde apontados nos prontuários investigados, estão apresentados na Tabela 3.

**Tabela 3:** Distribuição das condições clínicas e do processo de atenção à saúde dos pacientes que sofreram quedas em unidades de pronto-socorro, no período 2013-2019, Belo Horizonte, Minas Gerais, Brasil

Variáveis	n	%
**Condições clínicas** Confusão mental/agitação psicomotora	37	41,6
Alteração da marcha	22	24,7
Alteração cognitiva	16	18,0
Depressão	14	15,7
Distúrbio de equilíbrio	13	14,6
Histórico de queda	10	11,2
Ansiedade	8	9,0
Comprometimento auditivo	5	5,6
Comprometimento visual	5	5,6
Demência	1	1,1
**Processo de atenção à saúde** Acesso venoso periférico	66	74,2
Cateter Vesical de Demora	5	5,6
Cateter Nasoenteral	2	2,2

Em relação à avaliação de predição de queda, observou-se que em 42,7% dos prontuários havia o registro desta na data de admissão do paciente. Em 47,2% foram localizados o dia da ocorrência do evento. Já em 3,4% dos documentos pesquisados não havia anotação sobre a avaliação.

Dentre as condutas realizadas pelos profissionais de saúde após a ocorrência da queda estãoa realização de tomografia de crânio (20,2%), a avaliação clínica (18,0%), as instruções sobreprevenção de queda (14,6%), contenção mecânica de membros superiores (9,0%) e punção de veia periférica (4,5%).

As complicações e desfechos da internação hospitalar dos pacientes que sofreram queda nas unidades de PS estudadas estão descritas na Tabela 4.

**Tabela 4:** Distribuição das complicações e desfechos de pacientes que sofreram quedas nas unidades de pronto-socorro, no período 2013-2019, Belo Horizonte, Minas Gerais, Brasil

Variáveis	n	%
**Complicações devido à queda** Escoriação	11	12,3
Ferimento corto-contuso	11	12,3
Traumatismo cranioencefálico leve	9	10,1
Hematoma	7	7,8
Óbito	5	5,6
Fratura	4	4,4
Traumatismo cranioencefálico moderado	2	2,2
Traumatismo cranioencefálico grave	1	1,2
**Desfecho** Alta	52	58,4
Encaminhamento ambulatorial	21	23,5
Transferência para outra instituição	9	10,1
Óbito por complicações da queda	5	5,6
Óbito por complicações clínicas	2	2,4

Quanto à distribuição do grau do dano na população estudada, nenhum registro apontou a ocorrência de dano grave. Em 14,6% prontuários analisados não houve o registro deste dado. Do restante, 50,0% pacientes não apresentaram dano, 31,5% tiveram dano leve, 11,8% dano moderado e 5,6% foram à óbito.

À regressão logística binária, pacientes que evoluíram com complicações advindas da queda tiveram 20 vezes mais chance de danos com necessidade de intervenção e pacientes do sexo feminino tiveram 6,25 (1/0,16) vezes menos chances de danos moderados, graves ou óbito (Tabela 5).

**Tabela 5:** Fatores associados à ocorrência de dano com necessidade de intervenção (dano moderado, grave ou óbito) de pacientes que sofreram quedas nas unidades de pronto-socorro, no período 2013-2019, Belo Horizonte, Minas Gerais, Brasil

Variável	OR^*^	IC^**^ 95% Inferior	IC^**^ 95% Superior	Valor de *p*
Complicações devido à queda	19.64	2.21	174.8	0.007
Sexo feminino	0.16	0.03	0.9	0.037

* OR - Odds Ratio;

** IC - Intervalo de confiança.

## DISCUSSÃO

Sobre o perfil sociodemográfico, houve maior frequência no sexo masculino. A queda nesse gênero ocorre pelo fato dos homens, geralmente, solicitarem menos ajuda/auxílio na realização de atividades diárias, mesmo inseridos em instituições de saúde^(15)^. Ademais, para pessoas deste gênero, estar doente denota fraqueza, e não alteração das condições de saúde^(16)^.

A população de 60 anos ou mais foi a mais acometida neste estudo. Estudo qualitativo desenvolvido no estado do Paraná que investigou os saberes de idosas sobre prevenção de quedas mostrou que as pesquisadas reconheceram que fatores como fraqueza, doenças crônicas, deficiências visuais e cognitivas adicionados às fragilidades e limitações inerentes ao envelhecer contribuem para a ocorrência de quedas^(17)^. Ressalta-se que fragilidades cognitivas presentes em pessoas idosas são fatores preditivos para quedas^(12)^. Vale ressaltar que o crescimento desse perfil populacional culminará no maior número de internações desses, corroborando com o aumento da ocorrência de quedas^(18)^.

Quanto os aspectos clínicos, verificou-se que pessoas que caíram em PS eram fumantes e faziam uso de bebida alcoólica. O álcool e o tabagismo são fatores que contribuem para a queda pois ocasionam a deterioração da massa óssea e do tônus muscular, resultando na maior rigidez dos músculos^(19)^. Além disso, o consumo de álcool deprime o sistema nervoso central, resultando na marcha atáxica, alteração do equilíbrio, perda de reflexos e confusão mental^(19)^.

No que diz respeito às comorbidades, a hipertensão arterial foi a mais presente, corroborando com os estudos realizados em PS^(5,9)^. A presença dessa patologia causa espessamento da parede arterial e disfunção endotelial, seguidas por aumento na rigidez e diminuição na complacência vascular. Ainda, fragmentação e desordem das fibras de elastina que são substituídas por colágeno, o que facilita a deposição de íons cálcio impactando no funcionamento osteoarticular^(20)^. Essas modificações comprometem o equilíbrio corporal, tornando as pessoas hipertensas mais susceptíveis à queda^(20)^.

Em relação ao período em que as quedas ocorreram, o vespertino e a madrugada foram os mais frequentes. Durante a madrugada há uma redução do número de profissionais à beira do leito e menos pessoas circulantes no contexto hospitalar, além de menor assistência direta junto ao paciente. Isso compromete a vigilância dos pacientes e, por conseguinte, aumenta o risco de quedas neste período^(6)^. A permanência de um acompanhante para aqueles com alto risco de quedas poderá ser uma estratégia preponderante para a prevenção do evento em PS^(21)^.

A queda foi mais frequente em pessoas que estavam deitadas em maca pelo fato de pacientes admitidos em PS serem acomodados, na grande maioria, neste tipo de mobiliário. Quando em uso desse, o paciente permanece na mesma posição, o que ocasiona rigidez e fraqueza muscular e o instiga a movimentar-se na maca, potencializando a queda^(13)^. Ainda, esse resultado pode estar relacionado ao fato de que há uma maior demanda por parte da população brasileira da estrutura terciária pública, aqui inserido o PS, considerado a porta de entrada neste nível de atenção à saúde. Outrossim, o estudo foi realizado em dois hospitais componentes na rede de atenção às urgências e emergências da capital mineira e região metropolitana^(21)^.

A agitação psicomotora/confusão mental é apontada na literatura como fator de risco para quedas de pacientes^(22)^. Pacientes internados em PS apresentam uma complexa condição clínica, com várias morbidades associadas que podem resultar na agitação psicomotora/confusão mental^(23)^. O ambiente dessas unidades, por vezes agitado, com excesso de conversas ou barulho, somados ao moroso processo de adaptação ao ambiente hospitalar, especialmente em idosos, pode causar agitação/confusão mental nos pacientes, aumentando o risco de queda^(16)^.

No que se refere à predição de quedas, a avaliação de risco de queda deve ser realizada na admissão do paciente, diariamente e até a alta da instituição de saúde^(11)^. No entanto, no presente estudo, verifica-se a existência de discrepâncias frente à literatura. Pesquisa exploratória realizada em 2016 em oito unidades de pronto atendimento paranaenses constatou que, em um universo de 377 pacientes, nenhum foi avaliado e tampouco sinalizado quanto ao risco de queda^(22)^. Ao considerar o perfil de pessoas atendidas em PS, bem como as principais demandas ali atendidas, é notória a realização da predição de quedas, tanto durante a permanência no setor de triagem quanto em outro momento, em prol de um planejamento diretivo e seguro na prevenção do evento.

No Brasil, a Escala de Queda de Morse (MFS) é amplamente utilizada e recomendada como preditora de risco de quedas^(2)^. Contudo, essa parece não contribuir para uma avaliação efetiva derisco de quedas e tampouco para a implementação de cuidados direcionados a pacientes internados neste setor. A aplicação da MFS em pronto-socorro deve ser discutida.

Após o evento queda, os pacientes foram submetidos à tomografia computadorizada, bem como avaliação clínica. A avaliação médica após o evento deve ser imediata, para determinar as condições de saúde, possíveis complicações e, se necessário, intervenções clínicas urgentes e emergenciais^(24)^. A biomecânica da queda causa impacto de partes do corpo contra a superfície e, conforme a força exercida, causa lesões e traumas que, por vezes, não apresenta sinais e sintomas. Portanto, a realização de exames de imagem, como a tomografia computadorizada, deve ser feita como complementação à avaliação médica^(25)^.

Ferimentos corto-contusos e escoriações foram as complicações mais frequentes no estudo. Pesquisa descritiva e retrospectiva norte-americana desenvolvida em um PS mostrou que, das 57 quedas, 5,4% resultaram em escoriação^(26)^. Outro estudo estadunidense feito nesse contexto evidenciou que, de 190 eventos, as feridas corto-contusas ocorreram em 17,9%. Essas lesões são as mais habituais pelo fato de, no momento da queda, as pessoas tentarem se proteger. Habitualmente, esses ferimentos são predominantes nos membros superiores e inferiores^(27)^.

No presente estudo, cinco pacientes foram a óbito pelo evento. Ressalta-se que a taxa de mortalidade de idoso por queda em hospitais hospitalares aumenta cerca de 15% ao ano^(28)^. Dados de 2019 do DATASUS mostram que 342 pacientes adultos morreram em hospitais brasileiros por queda do leito. Estudo feito no Distrito Federal identificou que, no período de 1996 a 2017, um total de 2.828 idosos faleceram em decorrência de queda. Desses, 94,8% ocorreram na atenção terciária^(29)^.

Sobre o registro de grau de dano, a maioria apresentou nenhum dano ou dano leve. Das notificações recebidas pelo órgão, em quase a totalidade (169.742 - 98,1%) havia o registro do grau de dano. O leve foi o mais frequente (86.178 - 49,8%) e o óbito (429 - 0,25%), o menos^(7)^. Infere-se ainda que o resultado alcançado pode estar relacionado à limitação do conhecimento de parte dos profissionais de saúde sobre o conceito de queda bem como seus possíveis danos de cunho psicológico e social.

A respeito da associação das variáveis sociodemográficas e clínicas com grau de dano, pacientes do sexo feminino tiveram menor ocorrência de danos com necessidade de intervenção (moderados, graves ou óbito). Entende-se que mulheres seguem melhor as orientações de saúde recebidas e que, quando necessário, buscam atendimento de forma mais precoce.

Já os pacientes que evoluíram com complicações em função da queda apresentam maior ocorrência de danos com necessidade de intervenção. Estudo mostrou que dano advindo de queda, nesse incluídos traumas cranianos, pode agravar a condição clínica do paciente, inclusive para desfechos irreversíveis^(30)^. Deste modo, mesmo que intervenções mínimas sejam necessárias, a queda impacta na condição de saúde do paciente durante a internação.

Espera-se junto ao paciente internado em PS o melhor atendimento e com grandes possibilidades de sobrevivência. Contudo, a ocorrência do evento evitável queda acarreta complicações antes não presentes e/ou esperadas. Assim, a prevenção de queda é um desafio a ser alcançado em prol da minimização de complicações e desfechos desfavoráveis e, por vezes, irreversíveis^(21)^.

Analisar o contexto das quedas em PS e suas nuances é de suma importância para prevenção de sua ocorrência, uma vez que os pacientes internados nesse setor apresentam condições clínicas complexas e agravamento em sua saúde, o que mostra a relevância deste estudo.

### Limitações do estudo

Dentre as limitações, pode-se citar a inexistência, até o momento, de instrumentos validados para avaliar a ocorrência de queda em PS. Outro ponto foi a ausência do registro dos itens grau de dano, tipo de queda e avaliação de predição de queda à admissão e no dia evento nos formulários de notificações de algumas instituições, levando a perda de registros e limitando a realização de uma análise estatística mais robusta. Ainda, a subnotificação do evento queda neste setor limitou o tamanho da população e, consequentemente, a generalização dos resultados.

### Contribuições para a área da Enfermagem, Saúde ou Política Pública

Acredita-se que os achados deste estudo trazem contribuições para o ensino, pesquisa e prática assistencial da Enfermagem. Para o ensino, que estimule discussões e ampliação do aprendizado de futuros enfermeiros quanto à prevenção de quedas, com especial atenção à população idosa, que foi a mais acometida pelo evento tem maior chance de ser inserida em PS.

Para a pesquisa, que instigue a realização de estudos com delineamentos mais robustos e que abordem sobre as melhores práticas de prevenção de quedas nesta unidade. Para a prática assistencial, subsidie a implementação de protocolos específicos de prevenção de queda em PS, em prol da realização de cuidados mais diretivos, seguros e consistentes em prol da prevenção de quedas nesse setor.

## CONCLUSÕES

O estudo permitiu identificar que as quedas em PS ocorreram, na maioria, no período vespertino, em pacientes do sexo masculino e idosos. Estar acomodado em maca e com quadro clínico de agitação psicomotora/confusão mental foram os fatores mais frequentes neste contexto. A avaliação de predição de quedas não é frequente na admissão do paciente no PS ou no dia da ocorrência do EA. A realização da tomografia computadorizada, a avaliação clínica e a orientação de prevenção de queda são as condutas mais aplicadas após à queda. Em relação às complicações, os ferimentos corto-contusos e as escoriações foram as mais presentes. A alta hospitalar predominou como desfecho, e cinco pacientes evoluíram a óbito devido ao evento adverso. Constatou-se que apresentar complicações devido à queda foi associado a graus de dano que demandam mais intervenções e a desfechos irreversíveis. Ser do sexo feminino foi associado à ocorrência de danos sem necessidade de intervenção.

A análise das quedas nesse contexto requer maior sensibilização e investimento no contexto terciário, em prol do fomento de estratégias baseadas em evidências visando a redução do evento ao mínimo aceitável.

## Data Availability

Os dados de pesquisa estão disponíveis em repositório: https://doi.org/10.48331/SCIELODATA.UI4WW6.
